# Protocol to test the utility of detergents for *E. coli* membrane protein extraction and delipidation

**DOI:** 10.1016/j.xpro.2023.102146

**Published:** 2023-03-17

**Authors:** Virginia Wycisk, Leonhard H. Urner

**Affiliations:** 1 TU Dortmund University, Department of Chemistry and Chemical Biology, Otto-Hahn-Str. 6, 44227 Dortmund, Germany

**Keywords:** Model Organisms, Molecular/Chemical Probes, Protein Biochemistry, Protein Expression and Purification, Mass Spectrometry

## Abstract

We present a protocol to evaluate the utility of detergents for purification and delipidation of *E. coli* membrane proteins. We determine the critical aggregation concentration of detergents. Furthermore, we compare the ability of detergents to extract membrane proteins and to maintain protein-lipid interactions during purification. The protocol describes steps for isolating and delipidating membrane proteins from *E. coli* membranes by extraction and affinity purification using detergents. The protocol does not enable an absolute quantification of purification outcomes.

For complete details on the use and execution of this protocol, please refer to Urner et al.^1^

## Highlights

• Methodic screening of detergents for the purification of membrane proteins
• Understanding the aggregation behavior of a detergent
• Extraction and affinity purification of membrane proteins
• Estimation of relative delipidating properties of detergents by native MS

## Abstract (editor highlights)

Publisher’s note: Undertaking any experimental protocol requires adherence to local institutional guidelines for laboratory safety and ethics.

## Graphical abstract

**Figure:**
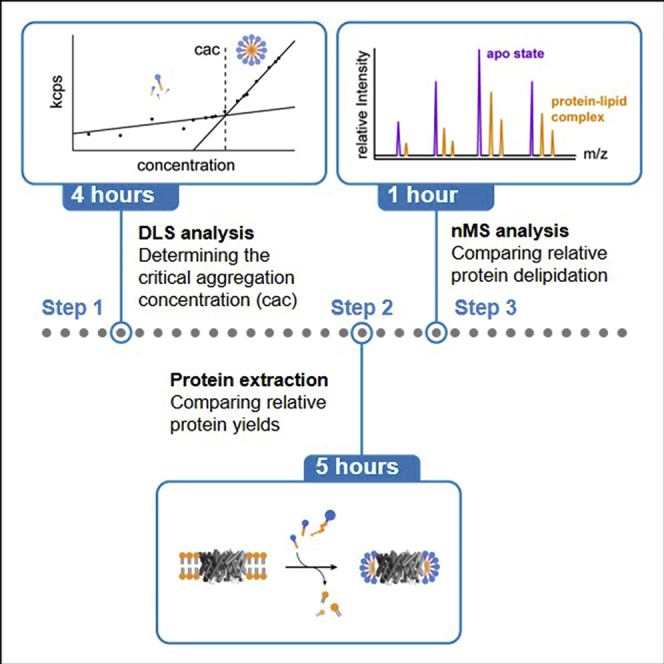

## Before you begin

Detergents can be used to extract proteins from membranes. Whether it be commercially available or newly synthesized detergents, this protocol gives you a step-by-step analysis on how to test the utility of detergents for the extraction and delipidation of *E. coli* membrane proteins.

Before starting with the protocol, detergents must be synthesized and purified. This protocol describes the use of a previously synthesized detergent, herein labeled as “hybrid detergent.” The hybrid detergent was purified by HPLC, and its structure was confirmed by NMR spectroscopy and high-resolution mass spectrometry. Further information on the synthesis and purification of the hybrid detergent can be found in this publication.^1^ Since this protocol aims for the assessment of the relative utilities of detergents for membrane protein purification, data will be compared with reference detergents. The aim of the here-chosen hybrid detergent example was to understand how combining the head groups of *n*-dodecyl-ß-D-maltoside (DDM) and tetraethylene glycol monooctyl ether (C8E4) into a hybrid detergent affects relative yields and delipidation obtainable from E. coli membrane proteins upon extraction and affinity purification.^1^ Therefore, DDM and C8E4 were included as commercially available references in this protocol.

This protocol also includes an experiment for the determination of the minimum information required from a detergent to test its utility for membrane protein purification and analysis: the critical aggregation concentration (cac).^2^ The cac is the minimum concentration of a detergent required to form aggregates in solution. To prevent membrane protein precipitation in the absence of membranes, detergent concentrations in purification buffers are traditionally adjusted to a multiple of the detergent’s cac.^3^ The experimental procedure for the determination of the cac was established based on the procedure described by Skhiri et al.^4^

Furthermore, before starting with membrane protein purification, *E. coli* membrane suspensions containing the overexpressed target membrane protein of interest are prepared. The target membrane protein is overexpressed by genetically modified *E. coli*. Membranes containing the overexpressed membrane protein of interest are separated from *E. coli*, concentrated, and suspended according to established protocols.^1^^,^^5^^,^^6^ Here, a His-tagged AqpZ-GFP construct was used as model system,^7^ since this membrane protein construct expresses very well, and the related purification workflow is well established and understood.^1^^,^^5^^,^^6^^,^^7^^,^^8^^,^^9^ Concentrated membrane suspensions are flash frozen in liquid nitrogen upon preparation and stored at −80°C prior to use. All buffer and detergent solutions are prepared prior to use. For native mass spectrometry (nMS) experiments, the customer support of the respective nMS instrument or experts in the field can be contacted if further help with parameter settings is needed. The nMS instrument must be ready for use before purified membrane protein samples are prepared.

This protocol allows to address three questions that may be important for the establishment of a new detergent entering membrane protein research: i) What is the cac of the detergent? ii) How much membrane protein does the detergent extract from E. coli membranes compared to a reference detergent? iii) How much membrane protein delipidation occurs during extraction and affinity purification with the detergent compared to a reference detergent?

### Preparation of protein-containing *E. coli* membrane suspension

**Timing: 1 week**

1. Preparation of Agar plates.
  a. Mix ingredients for agar plates and autoclave the mixture.
  b. Let the solution cool down to 40°C.
  c. Add 100 μL of ampicillin stock.
  d. Pour autoclaved mixture into petri dishes and let the plates dry for 5 min.

***Note:*** The mixture of ingredients for agar plates is sufficient for 6 plates.

2. Transform plasmid encoding ampicillin resistance and His-tagged AqpZ-GFP into C43(DE3) *E. coli* cells.
  a. Add 1 μL of plasmid solution (concentration = 100 ng/μL) to a 50 μL aliquot of commercially available C43 (DE3) *E. coli* suspension in a sterile 1.5 mL Eppendorf tube.
  b. Incubate the mixture on ice for 30 min.
  c. Heat shock the mixture in a warm water bath at 42°C for 45 s.
  d. Cool the mixture on ice for 2 min.
  e. Add 450 μL of starter culture medium.
  f. Shake the mixture with 180 rpm at 37°C for 1 h.
  g. Spread 50 μL of the mixture on an agar plate with a L-shaped cell spreader.
  h. Store agar plate in an incubator at 37°C for 16 h.
  i. Proceed with step 3 if individual colonies are visible on agar.
3. Express target membrane protein AqpZ-GFP in C43(DE) *E. coli* cells.
  a. Add 5 μL of ampicillin stock to 5 mL of starter culture medium.
  b. Transfer five colonies from agar plate into 5 mL of starter culture medium.
  c. Shake starter culture medium with 180 rpm at 37°C for 8 h.
  d. Add 400 μL of ampicillin stock to 400 mL overnight culture medium.
  e. Transfer starter culture medium into 400 mL of overnight culture medium.
  f. Shake overnight culture medium with 180 rpm at 37°C for 16 h.
  g. Add 12 mL of ampicillin stock to 12 L of expression medium.
  h. Transfer overnight culture medium into 12 L of expression medium.
  i. Shake expression medium with 180 rpm at 37°C until an OD600 between 0.7 and 1.0 is reached.
  j. Add 12 mL of the isopropyl-ß-D-thiogalactopyranoside stock solution to 12 L of expression medium.
  k. Shake expression medium with 180 rpm at 37°C for 4 h.
  l. Centrifuge expression medium (5,000 × *g* for 10 min) and discard supernatant.
  m. Add 2 Roche protease inhibitor tablets to 100 mL suspension buffer.
  n. Homogenize cell pellets in 100 mL suspension buffer.
4. Prepare membrane suspensions.
  a. Lyse cells by passing them four times through a Microfluidizer with an operating pressure of 1,500 bar and at a temperature of 4°C.
  b. Clarify supernatant by centrifugation (20,000 × *g* at 4°C for 20 min).
  c. Isolate and centrifuge supernatant (125,000 × *g* at 4°C for 45 min).
  d. Discard the supernatant.
  e. Add 1 Roche protease inhibitor tablet to 50 mL suspension buffer.
  f. Homogenize membrane pellet with 10 mL suspension buffer.
  g. Separate membrane suspension into 2 mL aliquots.
  h. Flash freeze 2 mL membrane suspension aliquots in liquid nitrogen.
  i. Store for up to 2 years at −80°C.

### Preparation of buffer and detergent solutions

**Timing: 60 min**

5. Preparation of buffer solutions.
  a. Prepare the extraction buffer, wash buffer and elute buffer according to the recipe described in the materials section.
  b. To prepare the elute buffer, mix 9 volume parts of wash buffer (18 mL) with 1 part of elute buffer (2 mL).
    ***Note:*** 20 mL should be enough for testing 3 detergents for protein extraction. Adjust the volume according to the number of detergents tested in the major step 2 (Protein extraction (steps 4–8).
    **Table:** Wash elute buffer

Reagent	Parts	Amount
Wash buffer	9	18 mL
Elute buffer	1	2 mL
**Total**	**N/A**	**20 mL**

Store at 4°C for up to 1 month.
  c. Prepare a detergent stock solution of 10w% (w/v) of hybrid detergent in MilliQ and dissolve it by gently agitating the mixture between 20°C and 25°C for 16 h.
    **Table:** Hybrid detergent stock solution

Reagent	Final concentration	Amount
Hybrid detergent	10 mg/mL	10 mg
MilliQ	N/A	1 mL
**Total**	**N/A**	**1 mL**

Store at 4°C for up to 12 months.

    ***Note:*** The time to solubilize detergents in water might vary. Some detergents require a few hours to be completely solubilized in water. If the water solubility of detergents is lower than 10 w% (w/v), use the highest w% (w/v) possible for the preparation of stock solutions.
  d. Prepare stock solution of the reference detergents (*n*-dodecyl-ß-D-maltoside (DDM) and tetraethylene glycol monooctyl ether (C8E4)) as described in the previous step 5c.
6. Detergent-containing ammonium acetate buffer for nMS experiments.
  a. Prepare the ammonium acetate buffer solution according to the recipe shown in the materials section.
  b. Mix the buffer with your detergent stock from step 5c according to the table below and aim for 2× the cac of the hybrid detergent.

**Table:** 

Reagent	Final concentration	Amount
Hybrid detergent (10 mg/mL)	420 μM	7 μL
Ammonium acetate (200 mM)	200 mM	1,993 μL
**Total**	**N/A**	**2 mL**

Store at 4°C for up to 1 week.

    ***Note:*** If the cac of the detergent is not known, skip to major step 1 (determination of critical aggregation concentration) and measure the cac of your detergent.
  c. Prepare the ammonium acetate buffer solution with the reference detergents accordingly.

### Instrument setup

7. Setting up the mass spectrometer.
  a. Use a modified Q Exactive mass spectrometer or a similar instrument and set the parameters as shown in the table below:

**Table:** 

Parameter	Settings
5 injection flatapole	7.9 V
Inter flatapole lens	6.9 V
Bent flatapole	5.9 V
Transfer multipole	4 V
Capillary voltage	1.2 kV
Source temperature	50°C
Voltage applied to the C-trap entrance lens	5.8 V
HCD cell voltage	0 V
HCD cell pressure	9 × 10^-10^ mbar
Noise level parameter	3
Microscans	1–10
resolution	17,500
  b. Save the data as an ipr.file.
    ***Note:*** These parameters are defined in an .ipr file. For help with tuning .ipr files please contact the customer support of Thermo Fisher Scientific or experts in the field.

## Key resources table

**Table:** 

REAGENT or RESOURCE	SOURCE	IDENTIFIER
**Bacterial and virus strains**		

C43 (DE3) *E.coli*	Cambridge Bioscience, UK	CAT#60446-1

**Biological samples**		

His-tagged AgpZ-GFP plasmid	Provided by A. Laganowsky	N/A

**Chemicals, peptides, and recombinant proteins**		

PBS, pH 7, sterile-filtered, suitable for cell culture	Sigma Life Science	CAT#806552-500 mL
Tris(hydroxymethyl)aminomethan (TRIS, Trometamol) ACS, Reag. Ph. Eur.	Thermo Scientific	CAT#75825-36
Sodium chloride	Thermo Scientific	CAT#424290010
Imidazole	Thermo Scientific	CAT#A10221
Glycerol	Sigma-Aldrich	CAT#8.18709.1000
Ni NTA agarose	Qiagen GmbH	CAT#30210
Ammonium acetate solution (7.5 M)	Sigma-Aldrich	CAT#A2706-100ML
Tetraethylene glycol monooctyl ether (C8E4)	Glycon Biochchemicals GmbH	D20038
n-Dodecyl-β-D-maltoside (DDM)	Glycon Biochchemicals GmbH	D97002
Methanol	Honeywell	CAT# 24229
Agar-Agar	Carl Roth	CAT#2266.1
LB broth	Carl Roth	CAT#X968.2
Ampicillin	Thermo Fisher Scientific	CAT#J60977.06
Roche Protease Inhibitor (cOmplete™ ULTRA tablets)	Sigma-Aldrich	CAT#5892988001
MilliQ	Self-prepared	N/A
Isopropyl-ß-D-thiogalactopyranoside	Merck Millipore	CAT#1370640100

**Software and algorithms**		

Zetasizer Nano Software v3.30	Malvern	Instrument Software
Tune	Thermo Fisher Scientific	Instrument Software
Xcalibur V2.2	Thermo Fisher Scientific	Instrument Software
Origin V9.1	OriginLab	www.originlab.com

**Other**		

Eppendorf tubes (1.5 mL)	Eppendorf	CAT#3810X
Centrifuge tubes (50 mL)	Greiner Bio-One	CAT#227261
Centrifuge tubes (15 mL)	Greiner Bio-One	CAT#188271
Petri dishes without vents	Carl Roth	CAT#EN16.1
L-shaped cell spreader	Carl Roth	CAT#PC57.1
ZebaTM Spin Desalting columns (MWCO 7 kDa)	Thermo Fisher Scientific	CAT#89882
Microfluidizer	Microfluidics	M-110P
Table top centrifuge	Eppendorf	5424 R
NanoPhotometer	Implen	NP80 UV/Vis
Zetasizer	Malvern, UK	Nano-ZS ZEN3600
Syringe filter	Carl Roth	CAT#PA49.1
Polystyrene Semi-micro cuvettes	Brand	CAT#759015
Quartz Suprasil Semi-micro cuvette (width × length: 2 mm × 10 mm)	Hellma	CAT#104-B-10-40
Amicon 0.5 mL centrifugal filters (MWCO: 100 kDa)	Amicon	CAT# UFC510024
Q Exactive instrument hybrid quadrupole-Orbitrap mass spectrometer	Thermo Fisher Scientific	N/A

## Materials and equipment

**Table:** Agar plate composition

Reagent	Final concentration	Amount
LB broth	25 g/L	2.5 g
Agar	15 g/L	1.5 g
MilliQ	N/A	100 mL
**Total**	**N/A**	**100 mL**

***Note:*** We recommend using this solution directly after preparation.

**Table:** Ampicillin stock solution

Reagent	Final concentration	Amount
Ampicillin	100 mg/mL	1.3 g
MilliQ	N/A	13 mL
**Total**	**N/A**	**13 mL**

Store at 4°C for up to 1 day.

**Table:** Isopropyl-ß-D-thiogalactopyranoside stock solution

Reagent	Final concentration	Amount
Isopropyl-ß-D-thiogalactopyranoside	0.5 M	1.54 g
MilliQ	N/A	13 mL
**Total**	**N/A**	**13 mL**

***Note:*** We recommend using this solution immediately after preparation.

**Table:** Starter culture medium

Reagent	Final concentration	Amount
LB broth	25 g/L	0.156 g
MilliQ	N/A	6 mL
**Total**	**N/A**	**6 mL**

***Note:*** Sterilize the starter culture medium in an autoclave immediately after preparation. The medium can then be stored at 4°C for up to 1 week.

**Table:** Overnight culture medium

Reagent	Final concentration	Amount
LB broth	25 g/L	10 g
MilliQ	N/A	400 mL
**Total**	**N/A**	**400 mL**

***Note:*** Sterilize the overnight culture medium in an autoclave immediately after preparation. The medium can then be stored at 4°C for up to 1 week.

**Table:** Expression medium

Reagent	Final concentration	Amount
LB broth	25 g/L	300 g
MilliQ	N/A	12 L
**Total**	**N/A**	**12 L**

***Note:*** Sterilize the expression medium in an autoclave immediately after preparation. The medium can then be stored at 4°C for up to 1 week.

**Table:** **Suspension buffer** (pH = 8 at 22.5°C)

Reagent	Final concentration	Amount
Tris	20 mM	1.51 g
NaCl	300 nM	4.2 g
Glycerol	20% (v/v)	63 g
MilliQ	N/A	250 mL
**Total**	**N/A**	**250 mL**

Store at 4°C for up to 1 month.

***Note:*** There is a possibility of bacteria growing in the solution. If any sign of precipitation is observed, the suspension buffer should be discarded.

**Table:** Extraction Buffer (pH = 8 at 22.5°C)

Reagent	Final concentration	Amount
Tris	50 mM	3.02 g
NaCl	150 mM	4.38 g
Gylcerol	10% (v/v)	63 g
MilliQ	N/A	437 mL
**Total**	**N/A**	**500 mL**

Store at 4°C for up to 1 month.

***Note:*** There is a possibility of bacteria growing in the solution. If any sign of precipitation is observed, the extraction buffer should be discarded.

**Table:** Wash Buffer (pH = 8 at 22.5°C)

Reagent	Final concentration	Amount
Tris	50 mM	6.07 g
NaCl	100 mM	11.68 g
Imidazole	20 mM	1.36 g
Gylcerol	10% (v/v)	126 g
MilliQ	N/A	874 mL
**Total**	**N/A**	**1,000 mL**

Store at 4°C for up to 1 month.

**CAUTION:** Imidazole is toxic when swallowed, and can cause skin irritation and serious eye damage. It may also cause damage to the unborn child. Wear protective gloves and safety glasses at all times.

**Table:** Elute Buffer (pH = 8 at 22.5°C)

Reagent	Final concentration	Amount
Tris	50 mM	3.03 g
NaCl	100 mM	2.92 g
Imidazole	500 mM	17.02 g
Gylcerol	10% (v/v)	63 g
MilliQ	N/A	437 mL
**Total**	**N/A**	**500 mL**

Store at 4°C for up to 6 months.

**CAUTION:** Imidazole is toxic when swallowed, and can cause skin irritation and serious eye damage. It may also cause damage to the unborn child. Wear protective gloves and safety glasses at all times.

**Table:** Ammonium acetate buffer (pH = 6.9 at 22.5°C)

Reagent	Final concentration	Amount
Ammonium acetate (7.5 M)	200 mM	26 μL
MilliQ	N/A	1,000 μL
**Total**	**N/A**	**∼ 1 mL**

Store at 4°C for up to 1 month.

## Step-by-step method details

### Determination of critical aggregation concentration

**Timing: 4 h per detergent**

The minimum information from a detergent required for membrane protein purification is the cac. The cac of a detergent can be determined with dynamic light scattering (DLS) by monitoring the intensity of light that is scattered by detergent solutions. The intensity of light scattered from a sample solution has the unit kilo-counts per second (kcps). For detergent concentrations below cac, lower kcps values are obtained, which are similar to the kcps value of detergent-free water. For detergent concentrations above the cac, a steep increase in kcps values can be obtained. Therefore, plotting kcps values obtained from a broad range of detergent concentrations can enable extrapolating the cac of detergents.

1. Sample and instrument preparation.
  a. For cleaning and the preparation of stock solutions solvents, filter the solvents (methanol and MilliQ) through a syringe filter (regenerative cellulose, 0.22 μm) prior to the experiment.
    **CAUTION:** Methanol is volatile, flammable, and toxic. Keep away from heat, hot surfaces, sparks, open flames, and other ignition sources. Wear protective gloves, protective clothing and safety glasses. Use under a fume hood.
    ***Alternatives:*** Methanol can be substituted for the less toxic isopropanol to rinse the cuvette.
  b. Prepare a dilution series of hybrid detergent in filtered milliQ water with concentrations of detergent between 10^-8^ and 10^-2^ mol L^-1^.
    i. First, make the stock solution **A** and filter it through a syringe filter (see above).
    ii. Prepare stock solution **B** from stock solution **A.**
    iii. Proceed with the other stock solutions **C**-**F** accordingly to ensure accurate concentrations.
      ***Note:*** You can find the required volumes of detergent stock solutions and milliQ water for the dilution process in the table below:
      **Table:** Stock A dilution series

Final concentration (mg/mL)	Add stock	Volume (μL)	Add MilliQ (μL)
20 (= stock **A**)	120 mg hybrid detergent	–	6,000
18	**A**	900	100
16	**A**	800	200
14	**A**	700	300
12	**A**	600	400
10	**A**	500	500
8	**A**	400	600
6	**A**	300	700
4	**A**	200	800
2	**A**	100	900
      **Table:** Stock B dilution series

Final concentration (mg/mL)	Add stock	Volume (μL)	Add MilliQ(μL)
1 (= stock B)	**A**	200	3,800
0.8	**B**	800	200
0.6	**B**	600	400
0.4	**B**	400	600
0.2	**B**	200	800
      **Table:** Stock C dilution series

Final concentration (mg/mL)	Add stock	Volume (μL)	Add MilliQ (μL)
0.1 (Stock C)	**B**	300	2700
0.08	**C**	800	200
0.06	**C**	600	400
0.04	**C**	400	600
      **Table:** Stock D dilution series

Final concentration (mg/mL)	Add stock	Volume (μL)	Add MilliQ (μL)
0.01 (Stock D)	**C**	200	1800
      **Table:** Stock E dilution series

Final concentration (mg/mL)	Add stock	Volume (μL)	Add MilliQ (μL)
0.001 (Stock E)	**D**	100	900
0.0001	**E**	100	900
  c. Rinse a Quartz Suprasil Semi-micro cuvette (width × length: 2 mm × 10 mm) with filtered methanol (3 × 1 mL) and filtered MilliQ (3 × 1 mL).
    **CAUTION:** Methanol is volatile, flammable, and toxic. Keep away from heat, hot surfaces, sparks, open flames, and other ignition sources. Wear protective gloves, protective clothing and safety glasses. Use under a fume hood.
    ***Alternatives:*** Methanol can be substituted for the less toxic isopropanol to rinse the cuvette.
  d. Turn on the Zetasizer Nano-ZS and allow the instrument to warm up for 30 min.
  e. Set the instrument parameters as follows:

**Table:** Zetasizer Instrument Parameters

Parameter	Settings
Material	Polystyrene latex
Dispersant	Water
Sample viscosity parameters	Dispersant viscosity as sample viscosity
Temperature	22.5°C
Equilibration time	120 s
Cell type	Quartz cuvettes
Measurement angle	173° Backscatter
Measurement duration	Manual
Number of runs	11
Run duration	10 s
Number of measurements	3
Delay between the measurements	0 s
Data processing	General purpose, normal resolution

**Figure 1:**
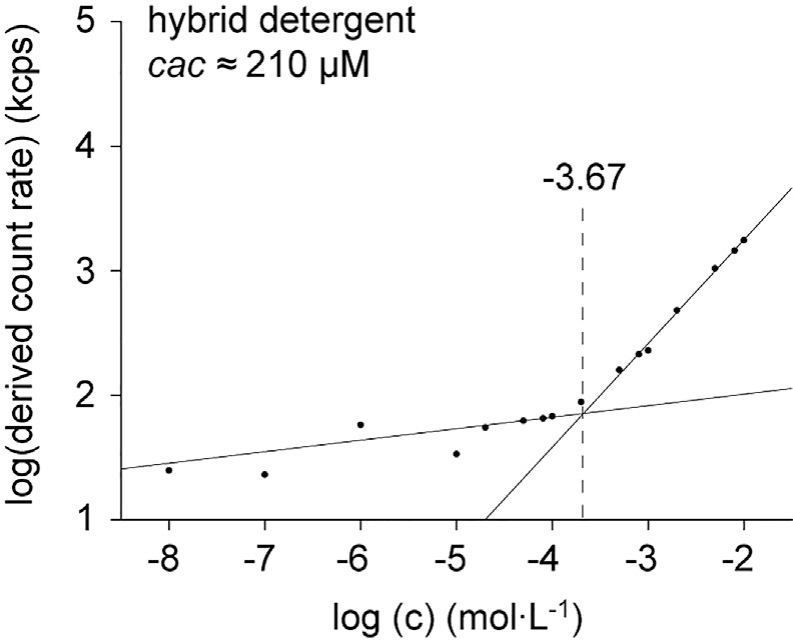
Determining the cac of detergents by DLS The logarithm of the derived count rate (kcps) is plotted against the logarithm of the detergent concentration. The flat region with low count rates at lower detergent concentrations and the linear growth of the count rate at higher detergents are fitted to linear functions. The intersection is takes as cac. (The image has been taken from (Urner et al.^1^) with the permission of the authors).

2. DLS Measurement.
  a. Use a glass Pasteur pipette to wash the cuvette with approximately 0.5 mL of the lowest concentration of detergent stock (0.0001 mg/mL). Discard the detergent solution.
    ***Note:*** This is a washing step. The cuvette is rinsed with the concentration to be measured so that any contaminations remaining are removed. The samples will be measured from lowest to highest concentration. Between the measurements the cuvette will be rinsed with the next higher concentration to ensure accurate measurements.
  b. Take a new Pasteur pipette and load approximately 0.5 mL of the solution containing the same detergent concentration (0.0001 mg/mL) into to the cuvette.
    ***Note:*** For accurate measurements the cuvette has to be filled so that the light beam passes through the solution. Here, we used 0.5 mL as minimum volume.
  c. Place the cuvette into the sample holder of the Zetasizer instrument.
  d. Start the measurement. Following the parameters defined in the software (see Table in step 1e).
    ***Note:*** The sample will be thermally equilibrated for 2 min at 22.5°C, and analyzed by three measurements, each containing 11 runs. Each run takes 10 s, which gives a measurement time of approximately 4 min per detergent stock concentration. The total measurement time is 85 min.
  e. Use a Pasteur pipette to remove the sample solution from the cuvette.
  f. Add approximately 0.5 mL of the next detergent stock concentration in the series (0.001 mg/mL) to the cuvette. Remove the solution from the cuvette.
    ***Note:*** This is a washing step. The cuvette is rinsed with the concentration to be measured so that any contaminations remaining are removed.
  g. Add the same concentration from the previous step 2f and apply steps 2c-e.
  h. Proceed with steps 2f and 2g and use the next higher concentration. Repeat until you reached the highest concentration (20 mg/mL).
    ***Note:*** Always start with the lowest concentration and gradually increase the concentration.
  i. After measuring the highest detergent concentration (20 mg/mL), rinse the cuvette with methanol (6 × 1.5 mL), MilliQ (3 × 1.5 mL) and methanol for a final rinse (1 × 1.5 mL).
    **CAUTION:** Methanol is volatile, flammable, and toxic. Keep away from heat, hot surfaces, sparks, open flames, and other ignition sources. Wear protective gloves, protective clothing, and safety glasses. Use under a fume hood.
    ***Alternatives:*** Methanol can be substituted for the less toxic isopropanol to rinse the cuvette.

3. Data processing.
  a. Calculate the average of the derived count rate values obtained from three measurements.
    ***Note:*** The unit of the derived count rate is kilo counts per second (kcps).
  b. Plot the logarithm of the derived count rate against the logarithm of the concentration.
    ***Note:*** The double logarithmic plots ideally show two characteristic regions: (1) a flat region with low count rates at lower detergent concentrations and (2) a linear growth of the count rate at higher detergent concentrations.
  c. Fit both regions to linear functions and take the concentration at the intersection as the cac value (Figure 1).
    **CRITICAL:** Measure detergent solutions in the order from lowest to highest detergent concentrations. Furthermore, the data output from Zetasizer Nano-ZS software can provide diffusion coefficients and hydrodynamic radii from any sample solution, regardless of the detergent concentration. The data related to detergent aggregates are most likely the ones obtainable above the cac.
    ***Optional:*** The measured sample can also be kept and used for other experiments.
    **Pause point:** The sample solutions used during DLS experiment are not crucial for the following protein purification experiments. The cac determination by means of DLS is over at this point and related detergent samples can be stored at 4°C for up to 12 months.

### Protein extraction

**Timing: 5 h for 20 protein-detergent combinations**

Detergents are used to extract membrane proteins from *E. coli* membrane suspensions. At this step, the utility of detergents to enable the extraction and affinity purification of His-tagged membrane proteins from *E. coli* membrane suspensions is evaluated. First, membrane suspensions are solubilized with detergent. Second, membrane proteins are purified by immobilized metal affinity chromatography (IMAC). Third, membrane proteins are eluted from IMAC columns and quantified by absorption spectroscopy.

4. Preparation of buffer solutions.
  a. Use the hybrid detergent stock solution made in step 4c (preparation of buffer and detergent solutions) and add it to wash, elute and wash elute buffer.
    ***Note:*** The final concentration of the detergent-containing buffer solutions should be 2× the cac of the detergent (here 420 μM).
    **Table:** Hybrid detergent-containing wash buffer

Reagent	Final concentration	Amount
Hybrid detergent (10 w% (w/v))	420 μM	17 μL
Wash buffer	N/A	4,983 μL
**Total**	**N/A**	**5 mL**

Store at 4°C for up to 1 week.
    **Table:** Hybrid detergent-containing elute buffer

Reagent	Final concentration	Amount
Hybrid detergent (10 w% (w/v))	420 μM	17 μL
Elute buffer	N/A	4,983 μL
**Total**	**N/A**	**5 mL**

Store at 4°C for up to 1 week.
    **Table:** Hybrid detergent-containing wash elute buffer

Reagent	Final concentration	Amount
Hybrid detergent (10 w% (w/v))	420 μM	34 μL
Wash elute	N/A	9,966 μL
**Total**	**N/A**	**10 mL**

Store at 4°C for up to 1 week.

    ***Note:*** It is recommended to use freshly prepared detergent-containing buffer solutions while the detergent-free buffer solutions can be stored for a month.
  b. Prepare the detergent-containing buffer solutions using the reference detergents according to the previous step 4a. Here, we used DDM and C8E4.
5. Preparation of protein.
  a. Cool all buffer and detergent solutions on ice.
  b. Put 900 μL extraction buffer to a centrifuge tube and add 100 μL of hybrid detergent stock solution (10 w% (w/v)) and add 100 μL of membrane suspension.
  c. Mix the solution by inverting them and cool the samples on ice for 10 min.
  d. Centrifuge the samples for supernatant clarification (12,000 × *g* at 4°C for 5 min).
6. Preparation of immobilized metal affinity chromatography (IMAC).
  a. Agitate bottle containing Ni-NTA agarose until it is a homogeneous suspension and add 800 μL of the Ni-NTA agarose suspension to empty spin columns.
  b. Remove the bottom caps of columns and apply pressure with your fingertip onto the upper opening of every column to initiate the solvent flow.
  c. Add 1 mL detergent-containing wash buffers and wait until they ran through the columns.
  d. Accelerate solvent flows by applying pressure with your fingertip onto the upper openings of the columns.
7. Immobilized metal affinity chromatography (IMAC) purification.
  a. Isolate clarified supernatants obtained from step 5f above and load them onto the columns.
    **CRITICAL:** Do not accelerate solvent flows by applying pressure with your fingertip onto the upper openings of the columns at this step.
    ***Note:*** Load comparable volumes of protein-containing supernatant for every detergent.
  b. Pass the following detergent-containing buffers through the columns to purify membrane proteins: 1 mL wash buffer, 4 mL wash elute buffer.
  c. Discard the flowthrough.
  d. Pass the following detergent-containing buffer through every column to elute the purified membrane proteins: 400 μL elute buffer.
  e. Collect the flowthrough.
8. Relative protein quantification.
  a. Collect the eluted sample in a polystyrene semi-micro cuvette (1 cm pathlength).
  b. Measure the absorbance values at 485 nm for every sample on a photo spectrometer (IMPLEN). Troubleshooting 1.
  c. Plot the absorbance values obtained at 485 nm against the detergents to compare relative protein yields.

***Optional:*** If samples are too diluted and no reliable absorption values at 485 nm can be determined in semi-micro cuvettes (1 cm pathlength), such as in the case of A485 values < 0.1, transfer all eluted membrane protein samples to Amicon 0.5 mL centrifugal filters with MWCOs that are lower than the molecular weight of the investigated membrane protein complex. Concentrate samples by centrifugation (12,000 × *g* at 4°C for 5 min). Determine concentrated sample volumes with an Eppendorf pipette. Determine the A485 value with a microvolume photo spectrometer (IMPLEN). Apply volume correction to the absorbance values: Divide the obtained sample volume by the largest sample volume detected among all samples from the same experiment. Multiply the result with the absorbance value detected at 485 nm. Apply this correction to all samples. Plot the A485 nm values against the detergent abbreviation to compare relative protein yields.

**Figure 2:**
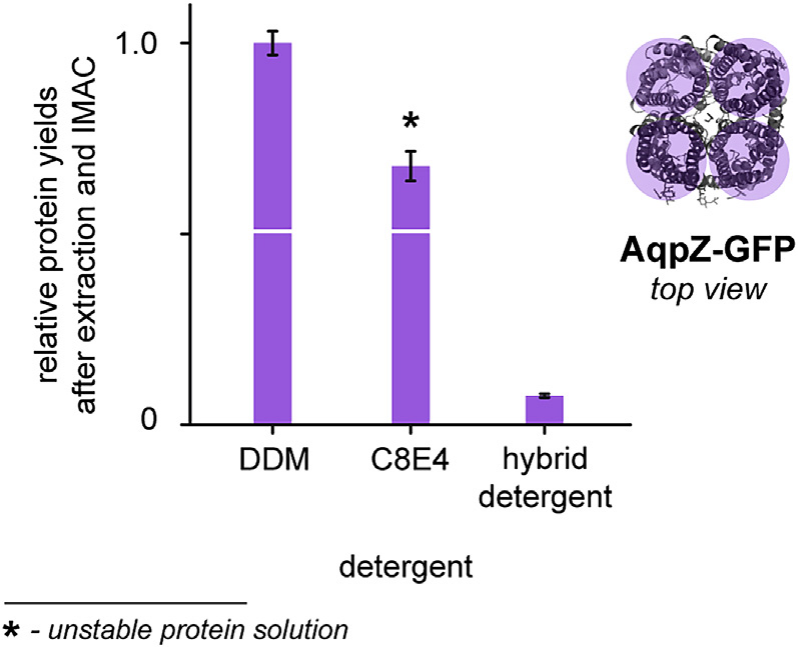
Comparing relative protein yields obtained from AqpZ-GFP in different detergents Higher relative protein yields are obtained from DDM and C8E4. However, membrane protein solutions obtained from C8E4 were not stable as indicated by protein precipitation during subsequent purification steps, e.g., concentrating or buffer exchange. Relative protein yields are shown with standard deviation from three repeats (n = 3). (The image has been taken from (Urner et al.^1^) with the permission of the authors).

***Note:*** Some detergents may enable the isolation of large relative protein quantities upon extraction and affinity purification but may lead to protein precipitation during subsequent purification steps, such as concentrating or buffer exchange. If this is the case, then label the relative protein yields with an asterisk to indicate that the obtained membrane protein solution may be not stable (Figure 2).

**Pause point:** The samples can be frozen in liquid nitrogen and stored at – 80°C for up to 6 months. The stability may depend on the individual protein. Before usage thaw the sample on ice.

### Native mass spectrometry

**Timing: 1 h**

Native mass spectrometry (nMS) is used to liberate the apo form of membrane proteins and membrane protein-lipid complexes from detergent micelles inside the vacuum of a mass spectrometer. nMS experiments performed under comparable conditions can be used to compare relative delipidating properties of detergents.

9. Sample preparation.
  a. Take 75 μL Zeba™ Spin Desalting columns (MWCO = 7 kDa) and remove lids from bottom and top of the column.
  b. Place the column into a 1.5 mL Eppendorf tube and remove shipping buffer by centrifugation (1,200 × *g* at 4°C for 1 min).
  c. Load the column with 50 μL detergent-containing ammonium acetate buffer and centrifuge (1,200 × *g* at 4°C for 1 min).
  d. Discard the eluted solvent.
  e. Repeat steps 9c-d four times in total.
  f. Load the column with 8 μL of concentrated protein solution, place the column into a fresh 1.5 mL Eppendorf tube, and centrifuge (1,200 × *g* at 4°C for 1 min).
  g. Store Eppendorf tubes containing the eluted membrane protein samples on ice.
  h. Dilute membrane protein-detergent mixtures with detergent-containing ammonium acetate to a comparable concentration, ideally a value between 1 – 10 μM.
10. Instrument preparation.
  a. Load .ipr file into the instrument software Tune.
  b. Load 2 μL of eluted protein solution into a gold-coated nMS capillary.^10^
  c. Place gold-coated nMS capillary into the nMS interface.
  d. In the instrument software, set HCD voltage to 0 V and capillary temperature to 50°C.
  e. Apply gas pressure to the capillary until a small bubble is formed at the front end of the capillary.
  f. Connect the nMS interface with the MS instrument.
  g. Switch on nMS voltage (capillary voltage).
  h. Acquire the mass spectra for approximately 5 s and save the data.
  i. Load MS data into the software Xcalibur, sum up the acquired mass spectra and check if protein signals are visible. Troubleshooting 2.
  j. Increase the HCD voltage by 50 V steps.
  k. At each HCD voltage change, acquire the spectra for 5 s and check if protein signals are visible by following the procedure described in step 10h above.

***Note:*** If protein signals are visible, identify the minimal HCD voltage required to obtain a resolved spectrum of your target protein with no detergents bound. Finish with a maximum HCD voltage of 300 V. It is recommended to scan the HCD voltage from 0 to 300 V to find the optimal conditions for the acquirement of membrane protein mass spectra. Troubleshooting 3.

**Figure 3:**
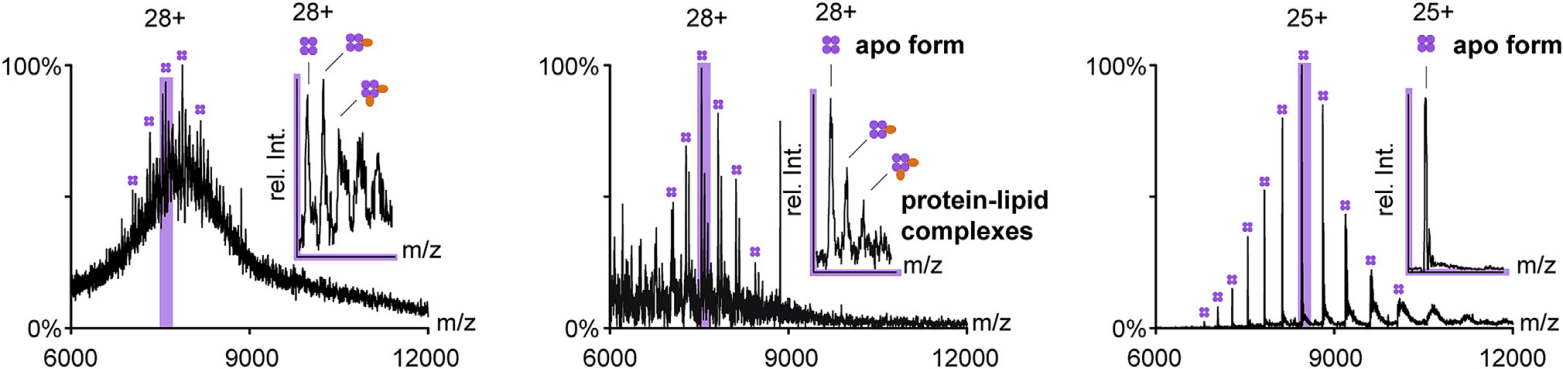
Comparing relative delipidating of AqpZ-GFP in different detergents by nMS Relative intensities of membrane protein apo form and membrane protein-lipid complexes are compared between different detergents. The relative intensity of membrane protein-lipid complexes decreases from DDM to hybrid detergent to C8E4. Samples were analyzed under comparable instrument conditions. (The image has been taken from Urner et al.^1^ with the permission of the authors).
  b. Assign membrane protein charge states by using MaCSED (For details and download see: http://benesch.chem.ox.ac.uk/resources.html) or Unidec.^11^
  c. Assign signals for membrane protein apo form and membrane protein-lipid complexes.
    ***Note:*** Compare the analyte mass obtained from MaCSED or Unidec with the theoretical protein mass calculated from its amino acid sequence or the sum of the theoretical protein mass and its ligand complexes.
  d. Compare similar charge states to assess relative trends in membrane protein delipidation.
    ***Alternatives:*** Extract relative intensities of membrane protein apo form and membrane protein-lipid complexes from nMS data and calculate the relative amounts for comparison.
    ***Note:*** To compare delipidating properties of detergents, extract relative intensities of apo form and protein-lipid complexes from mass spectra acquired at similar activation conditions, e.g., similar HCD voltage and capillary temperature. Latest instruments also allow additional ion activation through in-source trapping.

11. Data Analysis.
  a. Compare those mass spectra that were obtained from membrane proteins in different detergents and acquired using comparable protein concentrations and activation conditions (Figure 3).

## Expected outcomes

The protocol allows you to evaluate the general utility of a detergent for the extraction and affinity purification of membrane proteins from *E. coli* membrane suspensions. The first step of the protocol allows you to determine the cac of a detergent by means of DLS, which is the minimum information required for subsequent membrane protein tests. Furthermore, the DLS experiment provides additional information about the diffusion coefficient of detergent aggregates formed in water, their hydrodynamic radii, and whether the detergent forms monomodal or polymodal aggregate populations. This step can also be used to compare the cac values, diffusion coefficients, hydrodynamic radii, and polydispersity of detergents.

The second step of the protocol is used to investigate if detergents can be used to isolate large membrane protein quantities from *E. coli* membranes by extraction and affinity purification compared to reference detergents. This step can also be used to compare the relative utility of detergents to isolate membrane proteins from *E. coli* membranes by extraction and affinity purification (Figure 2). The absence of membrane protein precipitation during extraction and affinity purification can indicate that the membrane protein is stabilized in solution by the respective detergent.

The third step of the protocol is used to estimate the relative delipidating properties of detergents by nMS.

## Limitations

In the first step of this protocol, it can be difficult to obtain a scattering profile from the DLS experiment (see steps 3a-c) with detergents that have high cac values (>0.5 w% (w/v)), such as in the case of C8E4. The change in intensity of scattered light monitored by DLS is related to a change in refractive index of the sample caused by the formation of aggregates in solution. Detergent monomers also contribute to a change in refractive index of the sample. However, this usually becomes apparent at higher detergent concentrations. In case of detergents with high cac values, the increase in intensity of scattered light monitored by DLS can be affected by both high concentration of detergent monomers and the formation of aggregates. Therefore, a sharp transition between (a) a flat region with low count rates at lower detergent concentrations and (b) a linear growth of the count rate at higher detergents concentrations may not been obtained, thus rendering a determination of the cac by DLS difficult, such as in the case of C8E4.

The second step of the protocol is used to investigate if detergents can be used to isolate large membrane protein quantities from *E. coli* membranes by extraction and affinity purification. However, no information is obtained about whether the structure and function of membrane proteins are preserved by individual detergents. Furthermore, if protein yields are low, it remains unclear whether the detergent did not solubilize membranes and proteins or whether the detergent caused protein denaturation after solubilization.

The third step of the protocol is used to evaluate the relative delipidating properties of detergents by nMS. However, the protocol may not allow you to confirm the chemical identity of lipids or absolute quantification of co-purified lipids. Furthermore, it can be difficult to obtain mass spectra of intact membrane protein with detergents that co-purify large amounts of lipids.

## Troubleshooting

### Problem 1

The absorption of imidazole overlaps with the absorption of proteins around 280 nm. Therefore, a quantification of the protein may not be reliable at 280 nm (related to step 8b).

### Potential solution

Especially for non-GFP-tagged membrane proteins, do buffer exchange into imidazole-free buffer, for example, by using commercially available Micro Bio-Spin columns or desalting columns. Follow the manual for details on how to do the buffer exchange. Determine the absorbance at 280 nm.

### Problem 2

During nMS experiments no protein signals are visible (related to step 10i).

### Potential solution

First, check by eye if you have an electrospray and proceed with the following instructions: If no electrospray is present, disconnect the nMS interface from the MS instrument. Use a tweezer to clip a tiny bit of the gold-coated capillary off and repeat the steps 10c-i. Second, if electrospray is present but no protein signals are detected, increase the HCD voltage by 50 V steps. At each HCD voltage change, acquire the spectra for 5 s and check if protein signals are visible by following the procedure described in step 10j. If protein signals are visible, identify the minimal HCD voltage required to obtain a resolved spectrum of your target protein with no detergents bound. If protein signals are not visible, increase capillary temperature by 50°C steps and repeat HCD voltage ramps as described in step 10j.

### Problem 3

Many factors can result in a poor nMS spectrum, e.g., a) the membrane protein can aggregate inside the nMS capillary, b) the membrane protein may not be released from the micelle, c) the detergent may interfere with the nMS process, d) the membrane protein can dissociate inside the mass spectrometer, or e) trajectories of membrane protein ions do not reach the mass analyzer (related to step 10k).

### Potential solution

To validate a) the membrane protein aggregates, inspect the nMS capillary though the lens of a microscope. To validate the possibilities that b) the membrane protein may not be released from the micelle or c) the detergent interferes with the nMS process, include a protein-detergent combination that should work (positive control). To validate whether d) the intact membrane protein complex completely dissociates inside the mass spectrometer or e) trajectories of membrane protein ions do not reach the mass analyzer, scan the full set of parameters HCD voltages as described in steps 10d-j (step Native Mass Spectrometry). If applicable, include in-source activation voltage as additional parameter in your evaluation.

## Resource availability

### Lead contact

Further information and requests for resources and reagents should be directed to and will be fulfilled by the lead contact, Leonhard H. Urner (leonhard.urner@tu-dortmund.de).

### Materials availability

This study did not generate new materials.

## Data Availability

The published article (Urner et al.)^1^ includes all compounds generated or analyzed during this study.
